# Supplementary material for: Detailed molecular and epigenetic characterization of the pig IPEC-J2 and chicken SL-29 cell lines
Source: iScience. 2023 Feb 20;26(3):106252. doi: 10.1016/j.isci.2023.106252 (PMC10018572; doi:10.1016/j.isci.2023.106252)
Supplement: Data S1. Complete homer output for identified motifs in Pig IPECJ-2, related to Table 2 — Homer motif analysis results for histone modifications H3K4me1, H3K4me3, H3K27ac, and enhancer elements of pig IPECJ2 cell line. P-values >1e-10 are possible false positives. Within each folder (e.g. peak_files_CTCF) are the html files showing the identified motifs when using homer (e.g. homerResults.html). [file mmc2.zip › S5/Pig_IPECJ_2/peak_files_H3K4me3/homerResults/motif1.similar.html]

motif1

## Information for motif1

T
C
G
A
C
G
T
A
G
C
T
A
G
C
A
T
G
C
A
T
A
T
G
C
T
A
G
C
A
T
C
G
  
Reverse Opposite:  

A
T
G
C
A
T
C
G
T
A
C
G
C
G
T
A
C
G
T
A
C
G
A
T
G
C
A
T
A
G
C
T
  

|  |  |
| --- | --- |
| p-value: | 1e-316 |
| log p-value: | -7.278e+02 |
| Information Content per bp: | 1.486 |
| Number of Target Sequences with motif | 12129.0 |
| Percentage of Target Sequences with motif | 54.95% |
| Number of Background Sequences with motif | 11166.6 |
| Percentage of Background Sequences with motif | 42.20% |
| Average Position of motif in Targets | 150.6 +/- 86.9bp |
| Average Position of motif in Background | 148.2 +/- 104.0bp |
| Strand Bias (log2 ratio + to - strand density) | 0.0 |
| Multiplicity (# of sites on avg that occur together) | 1.33 |
| Motif File: | file (matrix) reverse opposite |

### Similar de novo motifs found

|  |  |  |  |  |  |  |  |
| --- | --- | --- | --- | --- | --- | --- | --- |
| Rank | Match Score | Redundant Motif | P-value | log P-value | % of Targets | % of Background | Motif file |
| 1 | 0.686 | G A T C A T C G A T G C G C T A A T C G G C T A C G A T G C A T | 1e-209 | -482.123066 | 73.62% | 63.86% | motif file (matrix) |
| 2 | 0.633 | T C A G C A T G G C A T G T A C A T G C T A C G A C T G T G C A | 1e-175 | -403.418688 | 45.78% | 36.49% | motif file (matrix) |
| 3 | 0.705 | C G T A G T A C C G T A C A G T G C T A C G A T G A T C A T C G G C A T A T G C G T A C C G T A C G T A | 1e-154 | -355.242682 | 56.08% | 47.16% | motif file (matrix) |
| 4 | 0.615 | T G C A C T G A C T G A C G A T A G T C A T C G T C A G A G C T | 1e-125 | -288.573675 | 20.72% | 14.74% | motif file (matrix) |
| 5 | 0.606 | T G A C A C T G C A T G A C G T C G A T A C G T T C G A T A G C | 1e-108 | -248.971091 | 22.88% | 17.05% | motif file (matrix) |
| 6 | 0.615 | T A G C C T A G C A G T G C T A G C T A C A T G C A T G G A T C C T A G G A T C A T G C A G C T G T A C | 1e-98 | -226.731797 | 65.40% | 58.46% | motif file (matrix) |
